# Norovirus Binding to Intestinal Epithelial Cells Is Independent of Histo-Blood Group Antigens

**DOI:** 10.1371/journal.pone.0066534

**Published:** 2013-06-14

**Authors:** Kosuke Murakami, Chie Kurihara, Tomoichiro Oka, Takashi Shimoike, Yoshiki Fujii, Reiko Takai-Todaka, YoungBin Park, Takaji Wakita, Tsukasa Matsuda, Ryota Hokari, Soichiro Miura, Kazuhiko Katayama

**Affiliations:** 1 Department of Virology II, National Institute of Infectious Diseases, Musashi-murayama, Tokyo, Japan; 2 Department of Internal Medicine, National Defense Medical College, Tokorozawa, Saitama, Japan; 3 Department of Applied Molecular Biosciences, Graduate School of Bioagricultural Sciences, Nagoya University, Furo-cho, Chikusa, Nagoya, Japan; Tulane University, United States of America

## Abstract

Human noroviruses (NoVs) are a major cause of non-bacterial gastroenteritis. Although histo-blood group antigens (HBGAs) have been implicated in the initial binding of NoV, the mechanism of that binding before internalization is not clear. To determine the involvement of NoVs and HBGAs in cell binding, we examined the localization of NoV virus-like particles (VLPs) and HBGAs in a human intestinal cell line and the human ileum biopsy specimens by immunofluorescence microscopy. The localizations of Ueno 7k VLPs (genogroup II.6) and each HBGA (type H1-, H2- and Le^b^-HBGAs) on the human intestinal cell line, Caco-2, were examined by confocal laser-scanning microscopy. To explore any interactions of NoVs and HBGAs *in vivo*, fresh biopsy specimens from human ileum were directly incubated with NoV VLPs and examined by immunofluorescence microscopy. We found that VLP binding depended on the state of cell differentiation, but not on the presence of HBGAs. In differentiated Caco-2 cells, we detected no type H1 HBGAs, but VLPs bound to the cells anyway. We incubated fresh biopsies of human ileum directly with VLPs, a model that better replicates the *in vivo* environment. VLPs mainly bound epithelial cells and goblet cells. Although the incubations were performed at 4°C to hinder internalization, VLPs were still detected inside cells. Our results suggest that VLPs utilize molecule(s) other than HBGAs during binding and internalization into cells.

## Introduction

Human noroviruses (NoVs) are members of the family *Caliciviridae* and the major cause of non-bacterial gastroenteritis worldwide [1]. Human NoVs are small non-enveloped viruses classified mostly into two main genogroups (GI and GII) [2], [3]. There are no vaccines or antiviral therapies to prevent or treat NoV infections. In addition, a lack of cell-culture systems or small-animal models for infection has hindered the study of the biology and pathogenesis of NoVs.

To explore the process of viral attachment to cells, we and others have used an experimental system of virus-like particles (VLPs) and the human intestinal cell line, Caco-2. Caco-2 cells were originally isolated from a human colorectal carcinoma [4]–[7]. They retain the ability to spontaneously differentiate into polarized, columnar cells with the characteristics of small intestine after reaching confluency [8]. VLPs self-assemble when the genes for the capsid protein are expressed in insect cells infected with a recombinant baculovirus [9]. These particles are thought to be morphologically and antigenically similar to native virions, and have been useful tools for studying virus-cell interactions [9]. VLPs of the prototype strain, Norwalk virus (GI.1), show increased binding to differentiated Caco-2 cells [7]. The C-terminal region (residues 300–384) of the major capsid protein VP1, which includes the histo-blood group antigens (HBGA) sites (site I, 325–331; site II, 340–346; site III, 373–381), is involved in cell binding [7], [10], [11]. In competition studies, a monoclonal antibody against the C-terminal region blocked its binding to Caco-2 cells [7].

Although the ligand(s) for productive NoV infection is unknown, type H HBGAs have been proposed as initial attachment factors [12], [13]. Thus, mutating the binding site abrogates binding to HBGAs [14]. HBGAs are complex carbohydrates that occur on mucosal epithelial cells or as free antigens in blood, saliva, and other secretions. Their core structures are classified into four major types, and they are converted into H antigenic structures by adding a fucose to a galactose (Gal) residue with an α1-2 linkage, catalyzed by the α1-2 fucosyltransferases, FUT1 and FUT2, in erythrocytes and in saliva and mucosal secretions, respectively [15]. The type 1 and type 2 core structures are converted into the type H1 and H2 HBGAs, respectively. Type H1 HBGA is further substituted on GlcNAc by a fucose in α1-4 linkage to yield type Le^b^ HBGA by α1-3/α1-4 fucosyltransferase, FUT3. Moreover, type H HBGAs are further modified on Gal by a GalNAc or a Gal in α1-3, resulting in type A and B HBGAs, respectively. This step is catalyzed by the A and B enzyme. Hemagglutination assays showed Norwalk VLPs bind to human type O and type A red blood cells specifically [16].

VLP binding to HBGAs can be strain-dependent. Several *in vitro* experiments based on enzyme-linked immunosorbent assays (ELISAs) characterized their interactions. *In vitro*, VLP-carbohydrate binding assays showed that Norwalk VLPs bound to type H1, H2, Le^b^- and Le^y^-synthetic saccharides [16]. In VLP-carbohydrate binding assays, VLPs of GII.6 Ueno 7k bound to type H2, H3, B, Le^a^, and Le^b^ synthetic carbohydrates. VLPs of another genotype, GII.1 (the 485 strain) failed to bind any HBGAs [17]. High-resolution X-ray structural imaging revealed that HBGAs bound to a surface-exposed shallow depression at the top of the P domain and that HBGA binding sites were different in viruses from GI and GII genotypes [10].

NoV VLP-HBGA binding has been well studied; however, the involvement and contribution of HBGAs in NoV and NoV-VLP binding to cells before internalization are not clear. Using confocal laser-scanning microscopy, we showed Ueno 7k VLPs (GII.6) specifically bound to a population of undifferentiated Caco-2 cells that formed clusters [4]. Interestingly, VLPs densely covered the entire surface of positive cells though they were not detected on neighboring cells [4]. More Norwalk VLPs bind to differentiated than undifferentiated Caco-2 cells [7]. Caco-2 cells express type H1 and Le^b^ HBGAs, depending on the state of cell differentiation [18]. Thus, HBGAs are likely to be involved in VLP binding to cells. Caco-2 cells are not productively infected by NoVs, suggesting a lack of factor(s) involved in infection, particularly in steps subsequent to cell-membrane binding. However, VLPs likely bind to Caco-2 by molecule(s) other than HBGAs. Studies involving VLPs of Ueno 7k showed binding to a 105-kDa protein in a virus overlay binding protein assay [5]. Therefore, we expected Caco-2 cells would provide us key information about mechanism(s) of NoV-cell binding/internalization

In this study, we investigated the co-localization of NoV VLPs and HBGAs to determine if expression of HBGAs has an effect on the binding of VLPs to Caco-2 cells. Expression of type H1, H2 and Le^b^ HBGAs was assessed on undifferentiated and differentiated Caco-2 cells, and binding of VLPs was determined by immunofluorescence. Furthermore, the involvement of HBGAs in binding of VLPs was evaluated in tissues from fresh ileal biopsies also by immunofluorescence.

## Materials and Methods

### Cell Culture

Caco-2 cells were obtained from the American Type Culture Collection (Rockville, MD) and maintained in Dulbecco's modified Eagle's medium (DMEM) supplemented with 10% fetal bovine serum (FBS) (Sigma, St. Louis, MO), 1% non-essential amino acids solution (Sigma), 100 U/ml penicillin and 100 µg/ml streptomycin, and grown under humidified 5% CO_2_ at 37°C. Cells were seeded at 50,000 cells/cm^2^ on type I collagen-coated coverslips (22-mm diameter, BD Bioscience, Bedford, MA) in six-well plates and on type I collagen-coated culture inserts in six-well plates (0.4-µm pore size, BD Bioscience). Cells on coverslips were cultured for 3 days and used as undifferentiated Caco-2 cells. Cells on culture inserts were cultured for 21 days and used as differentiated Caco-2 cells.

### VLP Preparation

VLPs of the Ueno 7k and 485 strains were prepared in a baculovirus expression system as described [19]. Briefly, a recombinant NoV VP1 capsid protein was expressed in the insect cell line High Five cells (Invitrogen, Carlsbad, CA), and VLPs secreted into the cell medium were collected by ultracentrifugation at 100,000×*g* in an SW32 rotor (Beckman, Palo Alto, CA). VLPs with the native virion size (i.e., 38-nm diameter) were purified by CsCl ultracentrifugation. Purified VLPs were applied to a carbon-coated electron microscopy grid, stained with 2% uranyl acetate, and examined by electron microscopy.

### Antibodies

Polyclonal antisera against Ueno 7k and 485 were obtained by immunizing rabbits with VLPs of Ueno 7k and 485, respectively [19]. The 5B18 mouse monoclonal antibody was obtained after immunization with GII.4 445 VLPs and is used as a GII broad-range capture antibody in a commercially available ELISA kit (Denkaseiken, Tokyo, Japan). Rabbit antibody against sucrase isomaltase and mouse monoclonal antibody against H2-HBGA (BRIC231) were purchased from Santa Cruz Biotechnology (Santa Cruz, CA). Mouse monoclonal antibodies against ZO-1 (ZO-1A12), H1-HBGA (BG-4) and Leb-HBGA (BG-6) were purchased from Invitrogen. Goat anti-mouse immunoglobulin conjugated to Alexa Fluor 488 and goat anti-rabbit immunoglobulin conjugated to Alexa Fluor 568 were used as secondary antibodies for immunofluorescence microscopy (Invitrogen). Goat anti-rabbit immunoglobulin conjugated to horseradish peroxidase (HRP) was used as secondary antibody for western blot (Thermo Fisher Scientific, Waltham, MA). Antibodies used in this study are summarized in Table 1.

**Table 1:** Antibodies used in this study.

Antibody	Clone	Species	Label	Application	Working concentration	Source
anti-Ueno 7k VLP	-	rabbit	-	WB/IF	1∶500	1
anti-485 VLP	-	rabbit	-	IF	1∶500	2
anti-GII NoV	5B18	mouse	-	IF	1∶500	Denkaseiken
anti-Sucrase isomaltase	-	rabbit	-	WB	1∶200	Santa Cruz
anti-ZO-1	ZO-1A12	mouse	-	IF	1∶400	Invitrogen
anti-H1-HBGA	BG-4	mouse	-	IF	1∶500	Invitrogen
anti-H2-HBGA	BRIC231	mouse	-	IF	1∶500	Santa Cruz
anti-Le^b^-HBGA	BG-6	mouse	-	IF	1∶500	Invitrogen
HRP-anti-rabbit IgG	-	goat	HRP	WB	1∶2,000	Thermo
Alexa Fluor-anti-mouse IgG	-	goat	Alexa488	IF	1∶1,000	Invitrogen
Alexa Fluor-anti-rabbit IgG	-	goat	Alexa568	IF	1∶1,000	Invitrogen

1 rabbit serum immunized with Ueno 7k VLPs [19].

2 rabbit serum immunized with 485 VLPs [19].

### SDS-PAGE and Immunoblotting

Sodium dodecyl sulfate–polyacrylamide gel electrophoresis (SDS-PAGE) was performed as described [20]. Protein samples were boiled for 3 min in SDS-PAGE sample buffer containing 2-mercaptoethanol. To estimate protein sizes, we used Precision Plus Protein Standards (Bio-Rad Laboratories, Hercules, CA) and visualized them with Coomassie brilliant blue R-250 (CBB) staining. Protein bands in SDS-PAGE gels were also detected by staining with CBB. For immunoblotting, proteins were blotted from the gel onto a polyvinylidene difluoride (PVDF) membrane and visualized with an enhanced chemiluminescence system (SuperSignal West Femto Maximum Sensitivity Substrate, Thermo Fisher Scientific).

### Immunofluorescence Microscopy of VLP-Cell Binding

Caco-2 cells cultured on coverslips or culture inserts were incubated with (+) and without (−) 0.5 µg of VLPs (equivalent to approximately 3.0×10^10^ particles) in 200 µl of DMEM at 4°C for 1 hour. After washing with DMEM without supplements three times, cells were fixed with 4% paraformaldehyde in PBS for 30 min at room temperature. After washing with PBS and blocking with NETG buffer (150 mM NaCl, 5 mM EDTA, 50 mM Tris-HCl, 0.05% Triton X-100, and 0.25% gelatin) for 2 hours at room temperature, cells were incubated with appropriate antibodies in NETG overnight at 4°C. Cells were subsequently incubated with Alexa dye–labeled antibodies in PBS for 1 hour at room temperature. Cell nuclei were stained with 4′6-diamidino-2-phenylindole (DAPI) (Dojindo Laboratory, Kumamoto, Japan). After washing with PBS, cells on coverslips or culture inserts were mounted onto glass slides in a drop of Prolong Gold anti-fade regent (Invitrogen). Imaging was performed on a Zeiss Axioplan2 microscope equipped with a LSM5 PASCAL laser-scanning confocal optics (Carl Zeiss, Thornwood, NY).

### Quantification of VLPs Bound to Caco-2 Cells

Nine microscopic images of Caco-2 cells were taken randomly at ×200 magnification (area of each image = 0.143 cm^2^). Areas of the red-channel signal corresponding to VLPs were measured by Photoshop software, version CS5 (Adobe Systems, San Jose, CA). Statistically significant differences between undifferentiated and differentiated Caco-2 were evaluated by Student's *t*-test. Error bars represent standard deviations, and statistical significance was defined as *p*<0.01.

### Immunofluorescence Microscopy of Biopsy Specimens

The study was approved by the Ethics Committee of the National Defense Medical College, and all biopsy samples were collected from two blood type O individuals (named as individual A and B in this study). Written informed consent was obtained from all donors. Biopsy specimens from human ileum were immediately cooled and incubated with 1.25 µg of VLPs (equivalent to approximately 7.5×10^10^ particles) in 500 µl of PBS(-) for 1 hour at 4°C. After washing three times with PBS(-), specimens were fixed with periodate lysine paraformaldehyde (10 mM NaIO_4_/75 mM lysine/2% paraformaldehyde) for 4 hours at 4°C. The specimens were dehydrated in a graded series of sucrose/PBS (10% for 4 h, 15% for 4 h, and 20% for 12 h, all at 4°C), embedded in OCT compound (Tissue Tek, Miles Laboratories, Elkhart, IN), and frozen at −80°C until use. Cryostat sections of 6 µm were incubated with primary antibodies and stained with the Alexa dye–conjugated secondary antibodies. Cell nuclei were stained with DAPI. Immunofluorescence images were obtained by confocal laser-scanning microscopy (Carl Zeiss). Pseudo-colors in the images, except for 5B18 detection, were changed by the Zeiss LSM Image Browser software (Carl Zeiss) as follows; the pseudo-color of the NoV VLPs, whose channel was red, was changed to green, and of each HBGA, whose channels were green, were changed to red.

## Results

### VLP-Cell Binding Was Greater in Differentiated than Undifferentiated Caco-2 Cells

**Figure 1:**
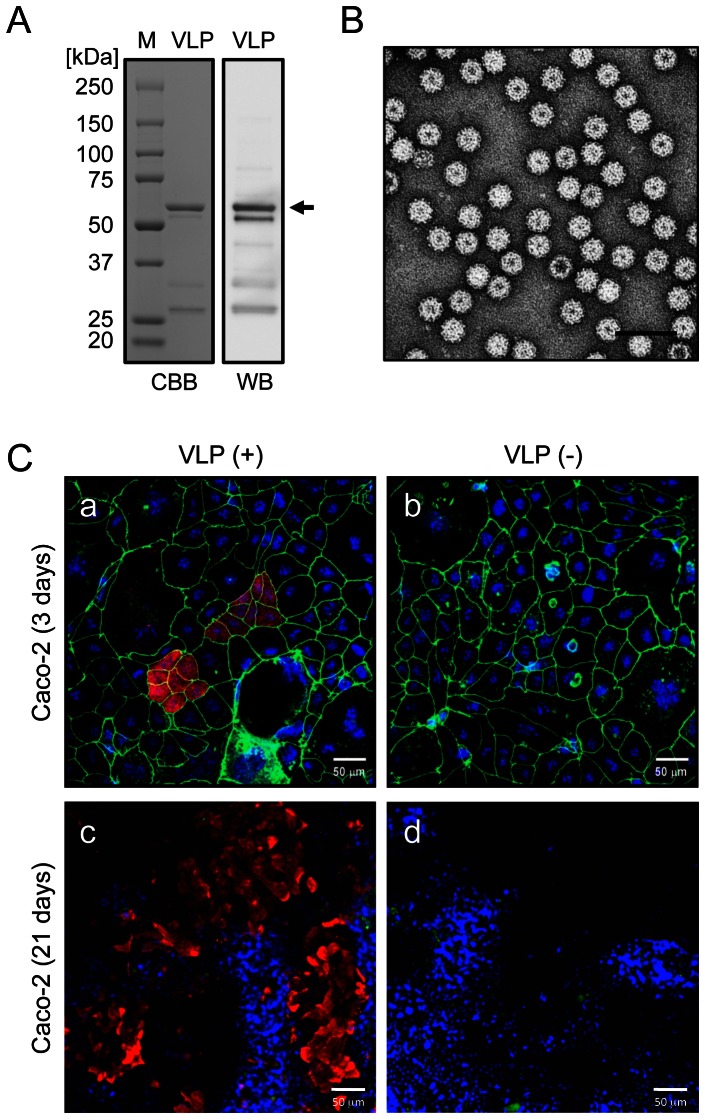
NoV VLPs specifically bound to undifferentiated and differentiated Caco-2 cells. VLPs of Ueno 7k strain were prepared in a baculovirus expression system. Purified VLPs were subjected to SDS-PAGE and examined by CBB staining (A, left) or western blotting with rabbit anti-Ueno 7k VLP serum (A, right). An arrow indicates the capsid protein, VP1. Purified VLPs were further examined by negative-stain electron microscopy (B). Scale bar in panel B = 100 nm. Caco-2 cells were incubated with (+) and without (−) 2.5 µg/ml of NoV VLPs at 4°C and fixed with paraformaldehyde. Cells were blocked and permeabilized with NETG and stained with rabbit anti-Ueno 7k VLP serum and mouse anti-ZO-1 antibody and subsequently with Alexa Fluor 568 goat anti-rabbit IgG, Alexa Fluor 488 goat anti-mouse IgG, and DAPI. Immunofluorescence micrographs were obtained by confocal laser-scanning microscopy (C). Red, NoV VLPs; green, ZO-1; blue, nuclei. Scale bars in panel C = 50 µm.

VLPs were purified by CsCl density gradient centrifugation and examined by SDS-PAGE, CBB staining, and western blotting with the anti-Ueno 7k VLP antibody (Fig. 1A). A major band was observed at 58 kDa, which corresponds to the major capsid protein VP1. Negative staining and electron microscopy revealed homogeneous particles with an expected diameter of 38 nm (Fig. 1B).

**Figure 2:**
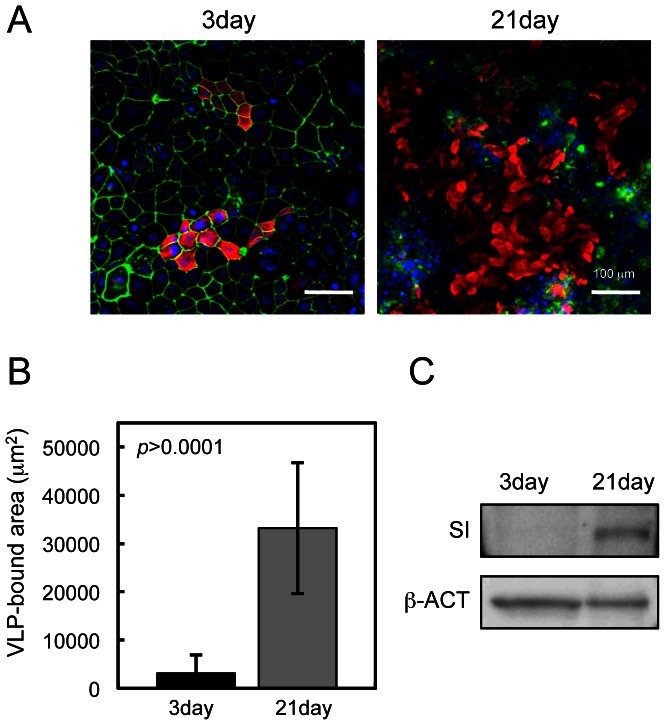
Binding of NoV VLPs to Caco-2 cells depended on the state of cell differentiation. Caco-2 cells were incubated with VLPs at 4°C and subjected to immunofluorescence microscopy (A). Areas of VLP binding on undifferentiated and differentiated Caco-2 cells were quantified with Adobe Photoshop software. Quantifications were performed with nine images, and Student's *t*-test was used for statistical comparisons. Results represent the means of ± S.D. of nine determinations (B). Caco-2 cells were lysed, and lysates were analyzed by SDS-PAGE/western blot with rabbit antibody against sucrase-isomaltase (SI), a differentiation marker for Caco-2 cells, with ß-actin as an internal control (ß-ACT) (C).

Undifferentiated Caco-2 cells were incubated with or without VLPs and examined by confocal laser-scanning microscopy. VLPs bound to a subset of cells that formed clusters, as reported [4] (Fig. 1C-a), and also bound to larger numbers of differentiated Caco-2 cells (Fig. 1C-c). No signals were observed in control cells incubated without VLPs (Fig. 1C-b and d). To investigate the effect of FBS on VLP-cell binding, undifferentiated or differentiated Caco-2 were incubated with VLP in PBS(-), and the results were the same as those in Fig. 1C (data not shown). To compare the amounts of VLPs on undifferentiated and differentiated Caco-2 cells, we measured the areas of VLP signals on immunofluorescent micrographs (Fig. 2A). The VLP binding areas on differentiated Caco-2 cells were approximately eightfold greater than those on undifferentiated Caco-2 cells (Fig. 2B). Levels of sucrase-isomaltase, a marker of intestinal differentiation, were higher in differentiated Caco-2 cells, confirming cell differentiation (Fig. 2C).

### VLPs Bind Undifferentiated and Differentiated Caco-2 Cells Independently of HBGA

**Figure 3:**
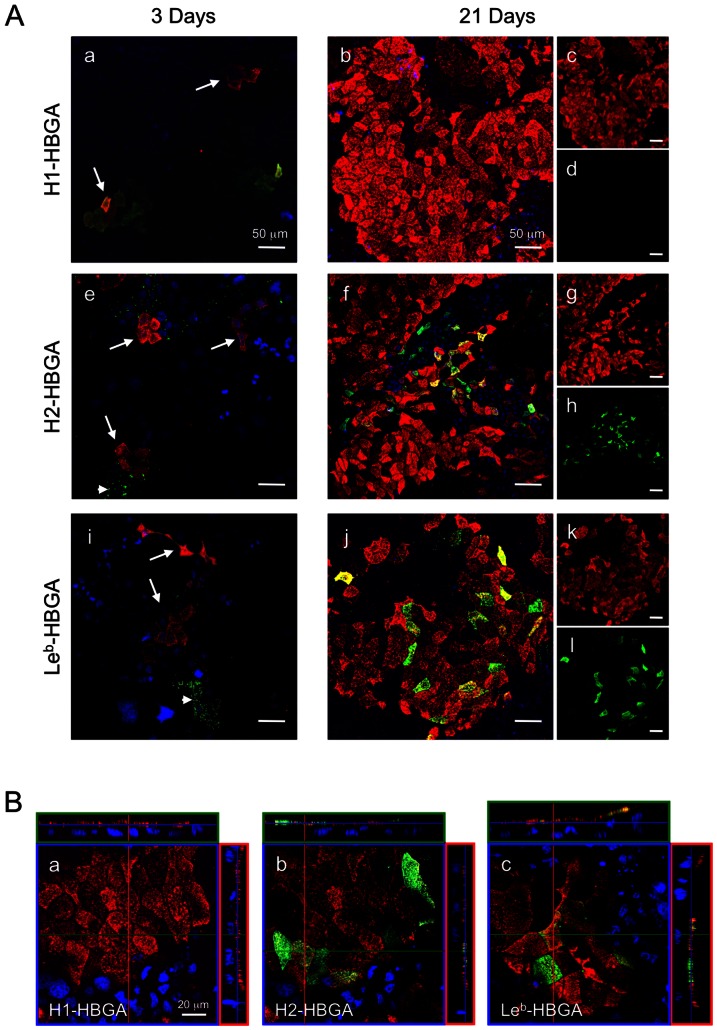
NoV VLPs bound to Caco-2 cells independently of type H1, H2 and Le^b^ HBGA expression. Caco-2 cells were incubated with VLPs at 4°C and fixed with paraformaldehyde. Cells were blocked and permeabilized with NETG and double-stained with rabbit anti-Ueno 7k VLP serum/mouse antibody against type H1, H2 or Le^b^ HBGA and subsequently with Alexa Fluor 568 goat anti-rabbit IgG/Alexa Fluor 488 goat anti-mouse IgG and DAPI. VLP binding is shown in the red channel (panels c, g, and k), type H1, H2, or Le^b^ are shown in the green channel (panels d, h, and l, respectively), colocalization of VLP binding and each HBGA is shown in a three-channel overlay (panel a, b, e, f, i, and j). Arrows indicate clusters of VLP binding cells in undifferentiated Caco-2 cells. Arrowheads indicate type H2 or Le^b^ HBGAs on undifferentiated Caco-2 cells (A). Typical Z-axis images of 21 day-cultured Caco-2 cells costained with NoV VLPs and type H1, H2 or Le^b^ HBGA were constructed digitally in the XZ/XY planes, shown as horizontal and vertical lines in the XY plane (B). Red, NoV VLPs; green, type H1 HBGAs, type H2 HBGAs or type Le^b^ HBGAs; blue, nuclei. Scale bars in panel A = 50 µm, and in panel B = 20 µm.

Confocal laser-scanning microscopy was used to determine the expression of HBGAs on Caco-2 cells and the correlation with VLP binding. HBGAs were seen on several clusters of undifferentiated Caco-2 cells. Type H1 HBGA was observed over the entire cell surface (Fig. 3A-a). Type H2 and Le^b^ HBGAs were seen in several populations in a dot-like pattern at cell membrane and in the cytosol (arrowheads in Fig. 3A-e and i). In undifferentiated Caco-2 cells stained for type H1, H2 and Le^b^ HBGAs, VLPs were observed bound to cells with or without HBGAs expression (arrows in Fig. 3A-a, e and i). In differentiated Caco-2 cells, type H2 and Le^b^ HBGAs were expressed over the entire apical surface of the majority of cells (Fig. 3A-h and l); however, type H1 HBGA was not detected (Fig. 3A-d). To confirm the possibility that type H1-antibody recognition could be blocked by VLP-cell binding, cells were preincubated with the antibody, and the result obtained was the same as shown in Fig. 3A-b (data not shown). VLPs bound mostly to differentiated Caco-2 cells (Fig. 3A-c, g and k), but binding was independent of HBGA expression (Fig. 3A-b, f, and j). To locate VLPs and each HBGA on differentiated Caco-2 cells in detail, cells were analyzed by three-dimensional imaging with confocal laser-scanning microscopy. Cross-sectional images showed that VLPs bound to the cells as a sheet covering the apical cell surface. Type H2 and Le^b^ HBGAs were detected on the apical cell surface, but type H1 HBGAs were not (Fig. 3B). On a subset of cells, type H2 and Le^b^ HBGAs were expressed on the apical surface of several cells and colocalized with VLP staining.

### VLPs Bound and Were Internalized into Intestinal Epithelium Independently of HBGAs

**Figure 4:**
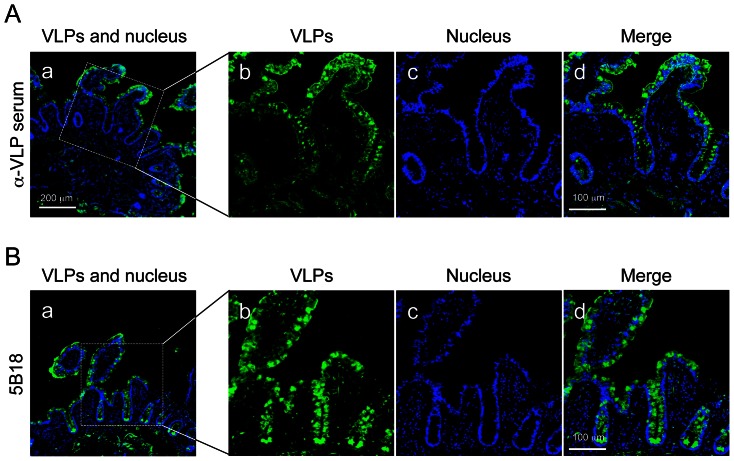
NoV VLPs specifically bound and internalized into human small intestinal epithelium. Fresh human ileum biopsy specimens from a single individual (individual A) were incubated with 2.5 µg/ml of VLPs in PBS(-) for 1 h at 4°C, and washed three times with PBS(-). After fixation with periodate lysine paraformaldehyde, specimens were dehydrated stepwise in 10, 15, and 20% sucrose/PBS, and embedded in OCT compound. Cryostat sections at 6 µm were incubated with primary antibodies specific for VLPs and stained with the Alexa dye–conjugated secondary antibody and DAPI, and subjected to confocal laser-scanning microscopy. The results of experiment with rabbit anti-Ueno 7k VLP serum and the 5B18 mouse monoclonal antibody as primary antibody are depicted in the panels A and B, respectively. Panels b–d are high magnification views of the area in boxes in panel a. Green, NoV VLPs; blue, nuclei. Scale bars in panel a = 200 µm, and in panel d = 100 µm.

**Figure 5:**
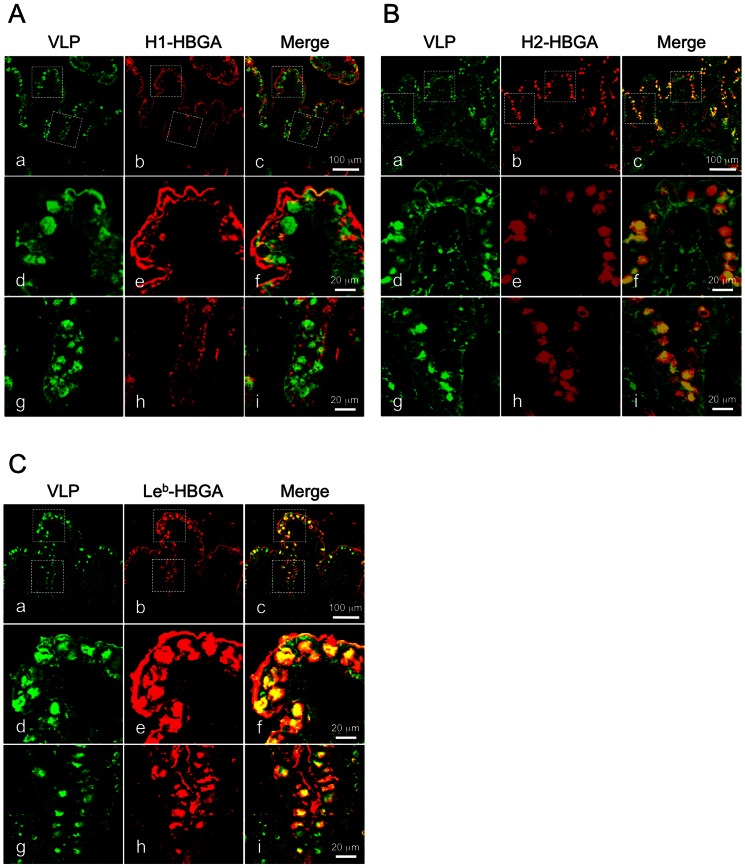
NoV VLPs were colocalized with each HBGA on the surface of epithelial and goblet cells. Fresh human ileum biopsy specimens from a single individual (individual B) were incubated with 2.5 µg/ml of NoV VLPs in PBS(-) for 1 h at 4°C and subjected to immunofluorescence microscopy. Cryostat sections were incubated with rabbit anti-Ueno 7k VLP serum and mouse anti-type H1, H2 or Le^b^ HBGA antibody and stained with Alexa dye–conjugated secondary antibodies and DAPI. Immunofluorescence images for type H1, H2 or Le^b^ HBGA are shown in panels A, B and C, respectively. Panels d–f and g–i are high magnification views of areas in boxes in panels a–c, respectively. Green, NoV VLPs; red, type H1 HBGA, type H2 HBGA or type Le^b^ HBGA. Scale bars in panel c = 100 µm, and in panel f and i = 20 µm.

**Figure 6:**
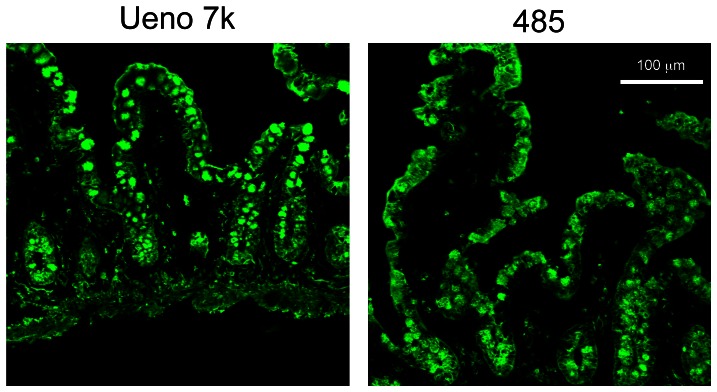
NoV VLPs of 485 strain also bound and internalized to intestinal epithelium. Fresh human ileum biopsy specimens from a single individual (individual B) were incubated with 2.5 µg/ml of Ueno 7k VLPs or 485 VLPs in PBS(-) for 1 h at 4°C and subjected to immunofluorescence microscopy. Cryostat sections were incubated with rabbit anti-Ueno 7k VLP serum or rabbit anti-485 VLP serum, and stained with Alexa dye–conjugated secondary antibody. Immunofluorescence images for Ueno 7k VLPs and 485 VLPs were depicted in left and right panels, respectively. Green, NoV VLPs. Scale bar = 100 µm.

To investigate the relationship of NoVs and HBGAs *in vivo*, we used immunofluorescence microscopy to examine VLP binding to fresh biopsies of human ileum. Fresh biopsy specimens from individuals with blood type O were incubated with VLPs at 4°C for 1 hour, fixed, embedded, cryo-sectioned, and examined. VLPs were detected in the intestinal epithelium, including epithelial and goblet cells, by a polyclonal rabbit antiserum against Ueno 7k VLPs (Fig. 4A). VLPs bound to the apical surface of epithelial cells on the villi and at the crypt level. To validate the specificity of the polyclonal antiserum in these biopsy specimens, we used the 5B18 IgG monoclonal antibody to detect the VLPs. This antibody has broad-range recognition of GII NoVs, and its binding to NoV VLPs has been well characterized by X-ray crystallography [21]. The use of the 5B18 yielded results comparable to polyclonal rabbit antiserum against Ueno 7k VLPs (Fig. 4B). We next double stained VLPs and each HBGA in the biopsy specimens to determine their localization. Type H1 HBGAs were preferentially detected on the apical surface of epithelial cells, and some were localized under the apical cell surface (e.g., in the fragmented layer). VLPs colocalized with type H1 HBGAs on the apical surface of epithelial cells (Fig. 5A). Type H2 HBGAs were strongly detected in goblet cells of intestinal epithelium, interestingly colocalizing with VLPs (Fig. 5B). Type Le^b^ HBGAs were found on the apical surface of epithelial cells and inside the goblet cells, and VLPs were colocalized at both sites (Fig. 5C). Since the biopsy specimens used in Figs. 4 and 5 were obtained from different individuals, we analyzed the localization of VLPs and type H2 HBGAs in specimens from both individuals. The samples showed comparable results (Fig. S1). The role of HBGAs in the binding and internalization of VLPs in biopsy specimens was evaluated with VLPs of the 485 strain, which do not bind HBGAs [17]. Results show binding and internalization into intestinal epithelium (Fig. 6, right), as well as Ueno 7k VLPs (Fig. 6, left).

## Discussion

In this study, we found that the NoV VLPs bind preferentially to differentiated Caco-2 cells and that this binding is irrespective of HBGA expression. Specifically, we found that type H1 HBGAs were expressed in some undifferentiated Caco-2 cells, but most VLPs bound to differentiated cells, which do not express the type H1 HBGAs (Fig. 3A-a to d). However, a small number of VLPs bound to cells that did not express type H1 HBGAs. A subset of undifferentiated and differentiated Caco-2 cells expressed type H2 and Le^b^ HBGAs, but VLP binding did not correlate with their expression (Fig. 3A-e to h, and -i to l, respectively). VLPs bound specifically to epithelial and goblet cells in fresh biopsies of human ileum. Although we performed the incubations at 4°C to prevent internalization, most VLPs were found inside of these cells. Type H1 and H2 HBGAs on the ileal specimens were observed primarily on the surface of epithelial cells and inside goblet cells, respectively. However, type Le^b^ HBGA was found at both sites. Type H1 and Le^b^ HBGAs colocalized on the surface of epithelial cells, and type H2 and Le^b^ HBGAs also colocalized in goblet cells, but not inside of epithelial cells. VLPs of the 485 strain, which do not bind HBGAs, were bound and internalized into intestinal epithelium, as well as Ueno 7k VLPs.

Caco-2 cells change morphological and functional characteristics remarkably during differentiation, and so, we estimated the effect of cell differentiation on the VLP binding. In previous studies of VLP-cell binding with the same cells and VLPs, approximately 5% of VLPs were internalized into cells at 37°C [5]. To separate binding and internalization, we incubated Caco-2 cells and VLPs at 4°C. We found that Ueno 7k VLPs bound intensely to differentiated Caco-2 cells (Fig. 2A), as reported for Norwalk and Ueno 7k VLPs [6], [7]. Cross-sectional images of differentiated Caco-2 cells showed the VLPs bound the apical surface and formed layers 2–4 µm thick (Fig. 3B). The numbers of VLPs added to 10^5^ cells in previous studies were 0.5–40 µg [7] (Norwalk) and 0–100 µg [5] and 1.5–15 µg [6] (Ueno 7k). Therefore, the ratio of VLPs and cells in the present study (0.25 µg VLPs/10^5^ cells) was thought to not be in excess. The VLP layers observed in differentiated Caco-2 cells might be due to the structure of the apical cell membrane. After differentiation, Caco-2 cells form microvilli on the apical surface, and the VLPs were assumed to bind the microvilli. The formation of microvilli is a likely reason for the thick VLP layer on Caco-2 cells. During cell differentiation, Caco-2 cells express many proteins on the microvilli surface that are mainly involved in xenobiotic and drug metabolism and lipid metabolism [22]. They might also be involved in VLP-cell binding.

In the present study, we found expression of type H2 and Le^b^, but not type H1, HBGAs (Fig. 3A). Our results differ from previous reports. For example, Amano *et al.* showed that the expression of type H1 HBGAs was increased during cell differentiation [18]. The reason for the differences is unknown. However, the exact levels of HBGA expression in Caco-2 cells are unclear. Only a few reports describe HBGA expression in Caco-2 cells during differentiation [18]. In addition, Caco-2 cells are a heterogeneous population and show appreciable differences in the transepithelial electrical resistance and permeability of hydrophilic marker substances in different laboratories [23].

Confocal laser-scanning microscopic analysis showed that Ueno 7k VLPs bound to undifferentiated and differentiated Caco-2 cells independently of HBGA expression. We wondered if VLP binding might hinder the binding of antibodies against HBGA. We preincubated Caco-2 cells with antibodies against HBGAs before adding VLPs and obtained the same images (data not shown). These results suggested the Ueno 7k VLPs rely on molecule(s) other than type H1, H2 and Le^b^ HBGAs to bind Caco-2 cells. Tamura *et al.* reported that Caco-2 cells have an unknown 105-kDa protein that was thought to be a candidate receptor molecule [5]. Therefore, we expected this molecule to be involved in VLP-cell binding. However, Marionneau *et al.* reported undifferentiated Caco-2 cells expressed type H3/4 HBGAs in addition to H2 HBGA. Thus, involvement of H3/4 HBGAs in VLP-cell binding should be investigated further.

We further examined the relationship of NoVs and HBGAs *in vivo* in ileum biopsies. Fresh biopsy specimens were incubated with VLPs at 4°C to suppress cellular uptake by phagocytosis, pinocytosis or endocytosis. Furthermore, we suspended the VLPs in PBS(-) to suppress cellular uptake activity, instead of DMEM/FBS which was thought to maintain that activity. The concentrations of VLPs (2.5 µg/ml) added to fresh biopsy samples were similar to those in previous studies (0.4–5 µg/ml) [24]–[26]. Surprisingly, VLPs were mainly detected inside epithelial cells and goblet cells, as well as on the surface of epithelial cells. Since an antibody might bind non-specifically, VLPs in biopsy specimens were stained with the 5B18 mouse monoclonal antibody and goat anti-mouse IgG conjugated with Alexa dye. The antibodies gave similar results (Fig. 4). The 5B18 monoclonal antibody broadly recognizes GII NoVs. X-ray crystallography showed it interacts with GII.10 at three sites, termed A, B, and C, in the P domain, in which six amino acids formed hydrogen bonds with amino acids of heavy chain. 5B18 binds highly conserved amino acids among other GII genotypes, including the GII.6 Ueno 7k strain [21]; therefore, 5B18 bound specifically to VLPs in the biopsy specimens. Non-specific binding of mouse IgG, as well as the fluorescence-labeled anti-mouse IgG, to the biopsy specimens could be ruled out, because each of the mouse IgG monoclonal antibodies to type H1, H2 and Le^b^ HBGAs gave different and characteristic staining images (Fig. 5 A–C).

Unlike previous studies of VLP-binding to intestinal biopsy [24]–[26], we used fresh biopsies to more accurately replicate the *in vivo* binding of NoVs. These samples might maintain cellular activity at 4°C, and thus, VLPs might internalize into epithelial cells and goblet cells. Internalization was fast and strong, and VLPs internalized into almost all cells. However, it was difficult to determine how the virus was internalized. Murine norovirus-1 (genogroup V) enters RAW264.7 macrophage cells by a non-clathrin, non-caveolae-, dynamin- and cholesterol-dependent pathway [27]. Feline calicivirus, a Calicivirus, enters Crandall Reese feline kidney cells by clathrin-mediated endocytosis and acidification into endosomes [28]. Thus, different members of the Calicivirus family enter cells by different mechanisms. We could not predict the cell entry pathway, but we expect VLPs were also internalized into cells by cell intake activity in addition to NoV-specific entry pathways because intestinal epithelial cells have strong uptake activity for nutrients.

Unexpectedly, VLPs were detected in goblet cells. This phenomenon was also observed in the pig duodenal specimens incubated with GI.1, GII.1, GII.3, and GII.4 NoV VLPs [24]. Although the strain of NoV VLPs used here was different from that of Cheetham *et al.*, the binding patterns of VLPs on the intestinal specimens were similar. Intestinal goblet cells synthesize and secrete high-molecular-weight mucus glycoproteins, mucins, that provide a viscous, presumably protective blanket on the mucosal surface. The synthesized mucins are stored within subcellular secretory vesicles, termed mucin granules, within goblet cells and secreted by exocytosis to the apical side of the cells. In the present study, mucin granule-like signals were clearly observed after staining with the H2- and Le^b^-antibodies (Fig. 5B and C), which presumably recognized the HBGA epitopes of goblet cell mucins. Moreover, VLPs signals co-localized to the area of mucin granules. Therefore, the VLPs detected in goblet cells might bind to mucin accumulated inside of the secretory granules of goblet cells.

However, the mechanism of internalization is still not clear because VLPs must move into the goblet cells against the flow of mucin secretion. VLPs might have been internalized into goblet cells by diffusion, because the activities of mucin synthesis and exocytotic secretion are decreased at 4°C. VLPs might have been internalized by endocytosis via still unidentified binding molecules expressed on the apical surface of enterocytes, as shown in this and previous studies, and the endosome-like vesicles containing VLPs might have fused with secretory vesicles containing mucins. Further studies are required to determine if such cellular processes are possible in biopsies of small intestine cooled at 4°C. VLPs may be taken up by enterocytes through a more specific mechanism, such as goblet-cell-associated antigen passages. These act as one of the antigen transcytosis systems in small intestine, by which intestinal goblet cells deliver low-molecular-weight soluble antigens from the intestinal lumen to underlying CD103^+^ dendritic cells and are also observed in human jejunum specimens [29]. Experimental infection studies of non-human primates included intestinal biopsy specimens from chimpanzees inoculated with Norwalk virus (GI.1) and from rhesus macaques inoculated with tulane virus (also known as rhesus enteric calicivirus). In these studies, viral antigens were detected in the lamina propria but not in enterocytes or goblet cells [30], [31]. Unlike our study, in which biopsy specimens were incubated with VLPs for 1 hour after VLP addition, biopsy specimens were obtained from monkeys 3–4 day after inoculations, and so, the locations of viral antigens might have changed, depending on infection stage.

The *in vitro* whole-virus binding assay, which was widely used to study the differential tropism characteristics of influenza viruses, also showed GII.4 VLPs bound to lamina propria and Brunner's glands in human duodenum [32]. However, biopsy specimens were formalin-fixed and paraffin-embedded and incubated with formalin-inactivated VLPs. Therefore, this assay might not accurately reflect viral binding/internalization to intestinal cells *in vivo*.

We found the Ueno 7k VLPs were likely to bind to type H1 and Le^b^ HBGAs on the surface of epithelial cells and to type H2 and Le^b^ HBGAs inside goblet cells. However, these results did not agree with those from ELISA-based assays, in which Ueno 7k VLPs bound to type H2, H3, B, Le^a^ and Le^b^ HBGAs [17]. Similarly, the VLP-HBGA binding pattern in biopsy specimens was different from that in the ELISA assays for other strains [25]. These results suggest that the VLP-HBGA assay *in vitro* does not completely reflect the binding mechanism *in vivo*. This hypothesis is strongly supported by our observation that the VLPs of 485 (GII.1) bound to ileum biopsy (Fig. 6). The previous study showed that VLPs of the same genotype of 485, Hawaii (GII.1), also bound to duodenal specimens of pig [24]. However, Hawaii VLPs were reported to bind to A, B and Le^b^ synthetic oligosaccharides [33], [34], although the reason for the different binding patterns of 485 and Hawaii was unknown. Therefore, the possibility remains that 485 VLPs bound to human ileum specimens by type Le^b^ HBGA, because Hawaii VLPs might bind to pig duodenal specimens by type A and/or Le^b^ HBGAs. However, the VLPs for HBGAs were thought to bind weakly, since Hawaii VLPs could not bind to any blood group saliva samples [34], which were more similar to the state of target *in vivo* than synthetic oligosaccharides. These VLPs might be supported by molecule(s) other than HBGAs for cell binding/internalization.

To clarify the involvement of HBGAs in VLP-cell binding/internalization, examination of the mutant VLPs, which are mutated in the HBGA-binding sites will be required. Tests of the GI.3 strains (Desert Shield and VA115), which were reported not to bind to any tested HBGAs, including human saliva samples [34], might also be helpful to understand the mechanism. The competition assays with monoclonal antibodies to HBGAs and the glycosidase depletion of HBGA structures in intestinal biopsy specimens also might be useful to investigate the involvement of HBGAs. However, these assays might injure the intestinal biopsy specimens because intestinal viability decreases immediately.

The results from previous studies that NoVs have the HBGA-binding site [7], [10], [11] and could not infect with non-secretors, which is the phenotype of the FUT2 inactivation [35], [36], demonstrate that HBGAs have an important role in Norovirus infections. However, a few strains (e.g., Desert Shield virus and VA115) could not bind to any HBGAs [34], and overexpression of FUT2 in Huh-7 cells was not sufficient to complete viral infection [37]. These results suggest that molecule(s) other than HBGAs are important for NoV binding to and internalization into cells. This hypothesis agrees with our observations that VLPs bound to Caco-2 cells independently of type H1, H2 and Le^b^ HBGAs. To our knowledge, this is the first report to incubate VLPs directly with fresh human intestinal biopsy specimens to examine VLP-cell binding. This result, that VLPs could bind and internalize to epithelial and goblet cells, might provide keys to disclose the mechanism of NoV infection.

## Supporting Information

Figure S1 **NoV VLPs colocalized with type H2 HBGA in intestinal biopsy specimens from a different individual.** Fresh human ileum biopsy specimens from a single individual (individual A) were incubated with 2.5 µg of NoV VLPs in PBS(-) for 1 h at 4°C and subjected to immunofluorescence microscopy. Cryostat sections were incubated with rabbit anti-Ueno 7k VLP serum and mouse anti-type H2 HBGA antibody and stained with Alexa dye–conjugated secondary antibodies. Panels d–f and g–i are high magnification views of areas in boxes in panels a–c, respectively. Green, NoV VLPs; red, type H2 HBGA. Scale bars in panel c = 100 µm, and in panel f and i = 20 µm.
(TIF)
