# Supplementary figures and images for: Norovirus Binding to Intestinal Epithelial Cells Is Independent of Histo-Blood Group Antigens
Source: PLoS One. 2013 Jun 14;8(6):e66534. doi: 10.1371/journal.pone.0066534 (PMC3682964; doi:10.1371/journal.pone.0066534)

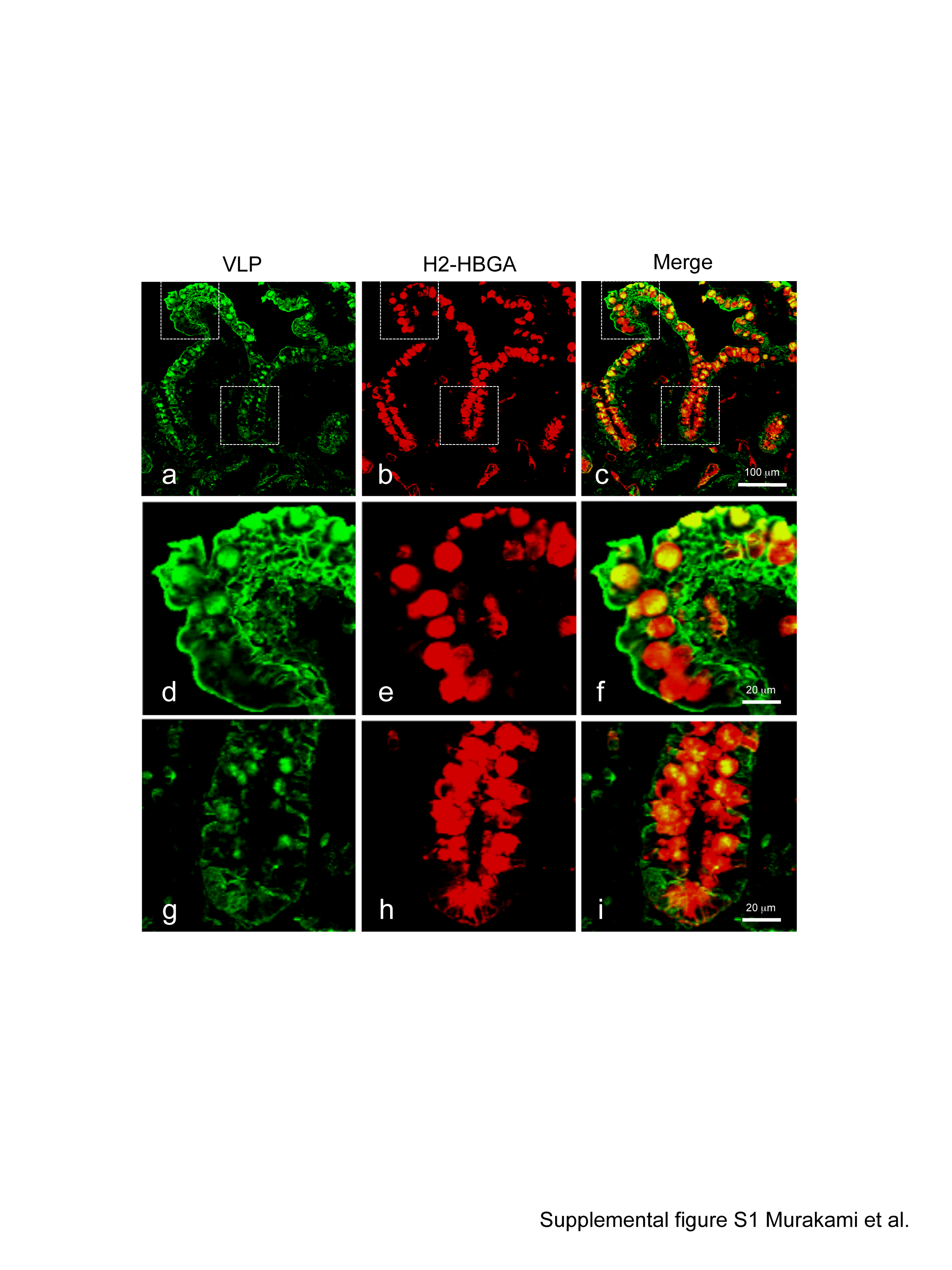

Supplement: Figure S1 — NoV VLPs colocalized with type H2 HBGA in intestinal biopsy specimens from a different individual. Fresh human ileum biopsy specimens from a single individual (individual A) were incubated with 2.5 µg of NoV VLPs in PBS(-) for 1 h at 4°C and subjected to immunofluorescence microscopy. Cryostat sections were incubated with rabbit anti-Ueno 7k VLP serum and mouse anti-type H2 HBGA antibody and stained with Alexa dye–conjugated secondary antibodies. Panels d–f and g–i are high magnification views of areas in boxes in panels a–c, respectively. Green, NoV VLPs; red, type H2 HBGA. Scale bars in panel c = 100 µm, and in panel f and i = 20 µm. (TIF) [file pone.0066534.s001.tif]
